# Empowering Researchers to Query Medical Data and Biospecimens by Ensuring Appropriate Usability of a Feasibility Tool: Evaluation Study

**DOI:** 10.2196/43782

**Published:** 2023-04-19

**Authors:** Christina Schüttler, Maria Zerlik, Julian Gruendner, Thomas Köhler, Lorenz Rosenau, Hans-Ulrich Prokosch, Brita Sedlmayr

**Affiliations:** 1 Central Biobank Erlangen University Hospital Erlangen Erlangen Germany; 2 Institute for Medical Informatics and Biometry Carl Gustav Carus Faculty of Medicine Technische Universität Dresden Dresden Germany; 3 Chair of Medical Informatics Friedrich-Alexander-Universität Erlangen-Nürnberg Erlangen Germany; 4 Federated Information Systems German Cancer Research Center Heidelberg Germany; 5 Complex Data Processing in Medical Informatics Medical Faculty Mannheim Mannheim Germany; 6 IT Center for Clinical Research University of Lübeck Lübeck Germany

**Keywords:** usability evaluation, ontology, feasibility queries, user-centered design, clinical research informatics, user interface

## Abstract

**Background:**

The *Aligning Biobanking and Data Integration Centers Efficiently* project aims to harmonize technologies and governance structures of German university hospitals and their biobanks to facilitate searching for patient data and biospecimens. The central element will be a feasibility tool for researchers to query the availability of samples and data to determine the feasibility of their study project.

**Objective:**

The objectives of the study were as follows: an evaluation of the overall user interface usability of the feasibility tool, the identification of critical usability issues, comprehensibility of the underlying ontology operability, and analysis of user feedback on additional functionalities. From these, recommendations for quality-of-use optimization, focusing on more intuitive usability, were derived.

**Methods:**

To achieve the study goal, an exploratory usability test consisting of 2 main parts was conducted. In the first part, the thinking aloud method (test participants express their thoughts aloud throughout their use of the tool) was complemented by a quantitative questionnaire. In the second part, the interview method was combined with supplementary mock-ups to collect users’ opinions on possible additional features.

**Results:**

The study cohort rated global usability of the feasibility tool based on the System Usability Scale with a good score of 81.25. The tasks assigned posed certain challenges. No participant was able to solve all tasks correctly. A detailed analysis showed that this was mostly because of minor issues. This impression was confirmed by the recorded statements, which described the tool as intuitive and user friendly. The feedback also provided useful insights regarding which critical usability problems occur and need to be addressed promptly.

**Conclusions:**

The findings indicate that the prototype of the *Aligning Biobanking and Data Integration Centers Efficiently* feasibility tool is headed in the right direction. Nevertheless, we see potential for optimization primarily in the display of the search functions, the unambiguous distinguishability of criteria, and the visibility of their associated classification system. Overall, it can be stated that the combination of different tools used to evaluate the feasibility tool provided a comprehensive picture of its usability.

## Introduction

### Background

The past decade has seen various projects aimed at making medical data and biological samples available for research. On a national level, the German Biobank Node (GBN) [1] pioneered biobanking, whereas the Medical Informatics Initiative (MII) [2] was able to establish infrastructure for processing and analyzing patient data from routine care by setting up data integration centers (DICs) at German university hospitals. In 2021, it was decided to merge these projects, which had previously run in parallel. The resulting project—*Aligning Biobanking and Data Integration Centers Efficiently* (ABIDE)—aims to harmonize technologies, regulations, committees, and governance structures of the 24 participating German university hospitals and their 25 biobanks to create a single point of contact for researchers searching for patient data and (associated) biospecimens. The central element will be a feasibility tool that researchers can use to query the availability of data and samples from routine care at the connected sites to determine the feasibility of their study project. The development of the tool should take into account that potential end users (laypersons) usually do not have specific knowledge regarding the execution of queries and that a too complex user interface, as found in, for example, expert tools such as ATLAS [3], should be avoided.

The ABIDE project benefits from previous work using the infrastructure of the DICs established within the MII. In addition, the ABIDE project takes advantage of the experience gained from the German Biobank Alliance (GBA) [4], which is coordinated by the GBN. Beyond this, the development work of the Network University Medicine COVID-19 Data Exchange Platform (CODEX) [5] project can be seamlessly integrated. In the CODEX project, based on prespecified requirements, a first test version of the envisaged feasibility tool (hereinafter referred to as feasibility tool v1) for simple queries has already been implemented and evaluated by potential end users regarding user-friendliness [6-8].

The feasibility tool v1 at the time allowed a simple querying of data elements based on the COVID-19–specific German Corona Consensus Data Set (GECCO) [9] and executing of federated queries on the Fast Healthcare Interoperability Resources (FHIR) servers at the MII DICs at the distributed sites. Data elements could be selected either via a free-text search field or a category tree and added as inclusion or exclusion criteria to a query. In addition, the criteria could be linked using Boolean operators. The usability analysis of the feasibility tool v1 showed that the previous developments were perceived as positive by users [8]. In particular, users found the intuitive operating concept convincing.

Nonetheless, some usability problems were uncovered. Among the points noted were a need for clearer visualization of the subdivision of inclusion and exclusion criteria, a uniform display of linking using Boolean operators, and the ability to search for synonyms. In addition, a function was desired to save a created query and continue editing it later or to archive sent queries together with the results. These and other functionalities were the focus of the development of an improved version of the feasibility tool in the ABIDE project (hereinafter referred to as feasibility tool v2) as additional requirements. In addition, focus was placed on the integration of the temporal restriction of criteria, grouping of criteria, and representation of their temporal relationship to each other, which was defined as an additional technical development goal for the ABIDE project. Another priority was the extension of the searchable data set. This was intended to expand the underlying ontology to the entire core data set of the MII [10], including biospecimens, so that it would no longer be limited to the GECCO.

In this way, the entire patient collective of the participating university hospitals can be considered in future study cohorts by means of appropriate feasibility queries. Furthermore, the integration of the feasibility tool v2 into the German Research Data Portal for Health (Forschungsdatenportal für Gesundheit [FDPG]) [11] will allow researchers to coordinate their research centrally via 1 platform.

The planned implementation was tested during development using a simulation prototype and supplementary mock-ups. On the basis of the feedback, a revised version of the feasibility tool (v3) will be created, which can then serve the development team as a reference for the final programming.

### Objectives

The objectives of the study were as follows: (1) an evaluation of overall user interface usability, (2) the identification of critical usability issues, (3) comprehensibility of the underlying ontology operability, and (4) analysis of user feedback on additional functionalities. From these, recommendations for quality-of-use optimization, focusing on more intuitive usability, were derived.

## Methods

### Study Design

We conducted an exploratory usability test consisting of two main parts:

1. The thinking aloud method, in which test participants express their thoughts aloud throughout their use of the tool, was complemented by a quantitative questionnaire.
2. The interview method was combined with supplementary mock-ups to collect users’ opinions on possible additional features.

The participants tested the feasibility tool v2 on the web from their workplace. Neither randomization into intervention and control groups nor blinding took place.

### Ethics Approval

The ethics committee at the Friedrich-Alexander-Universität Erlangen-Nürnberg approved the study (21-420-S).

### Recruitment

The focus of the study was on the primary user group of the feasibility tool v2. These are researchers who have a research question and require a cohort with specific patient data or available biospecimens to address it. Professionals with a biobanking background and IT specialists with a research background were also recruited. This is because they are considered a secondary user group as it can be assumed that they will also use the tool (eg, to process internal queries). Recruitment was initiated and coordinated by the ABIDE project management, and potential study participants were contacted through project staff at each site. One prerequisite was that the participants should have had no prior experience with the tool to be tested. This prevented an overlap with those who tested the first prototype. In accordance with the requirements of the study protocol, a sufficient number of individuals were approached to achieve the sample size of at least 14 volunteers.

### Description of the Feasibility Tool v2

**Figure 1:**
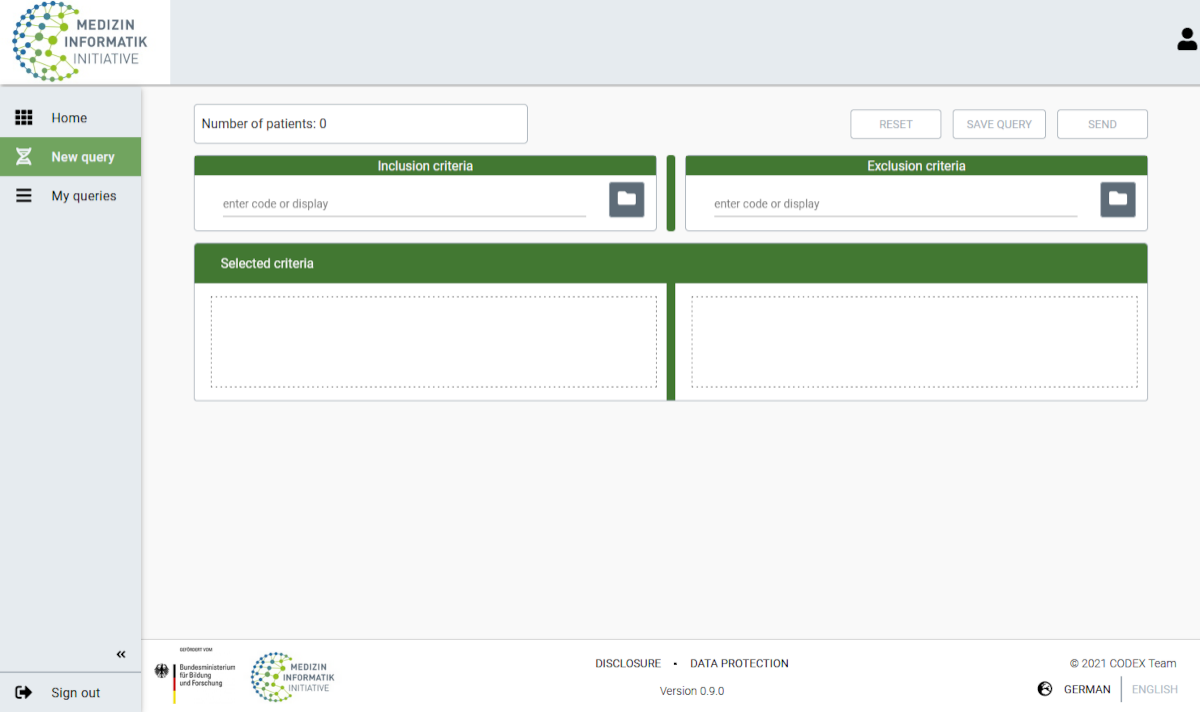
Search interface of the feasibility tool v2 of the *Aligning Biobanking and Data Integration Centers Efficiently* project.

The feasibility tool v2 was evaluated in January 2022. Compared with the first release, this version includes the core MII data set in addition to the GECCO. This enhancement means that the FDPG can ultimately serve as a central point of contact for people who want to check the Germany-wide availability of data and biospecimens from affiliated university hospitals to answer their research questions. In alignment with study protocols, in which exclusion and inclusion criteria are usually formulated for research questions, the interface of the feasibility tool v2 was designed to be structured accordingly (Figure 1).

**Figure 2:**
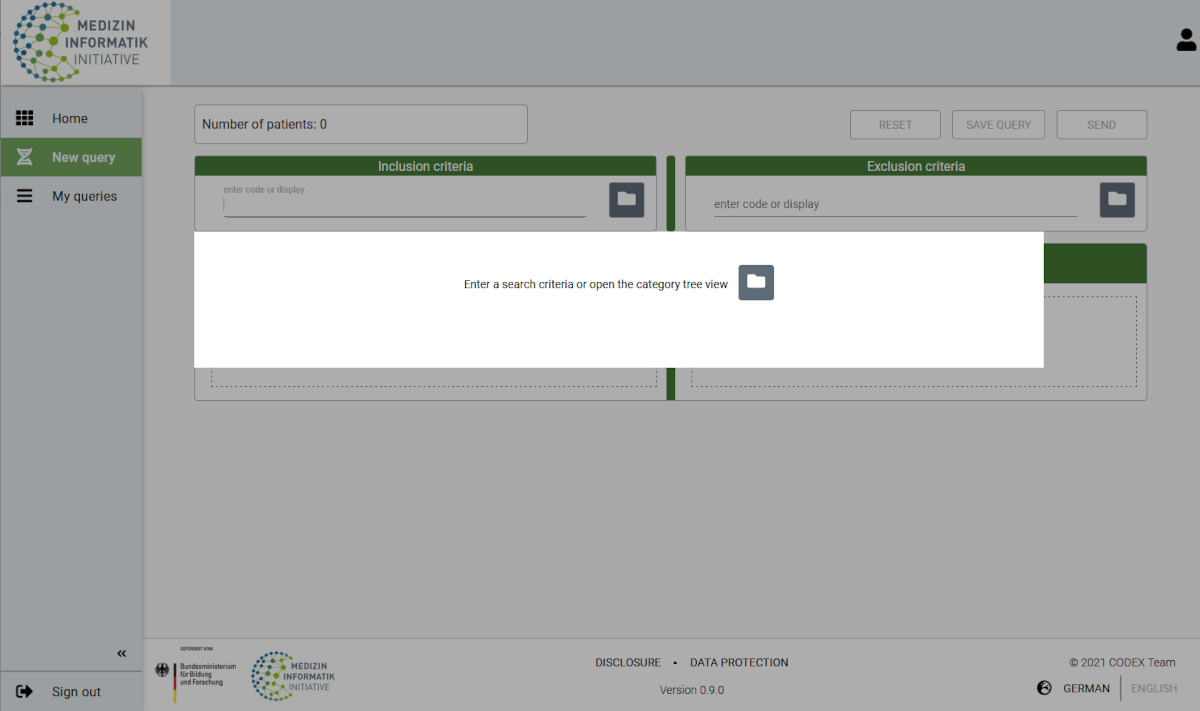
Search options via free-text search or category tree.

Criteria that are relevant for the study or should be avoided can be searched for in the respective areas using either a free-text search or a category tree (Figure 2) and selected.

**Figure 3:**
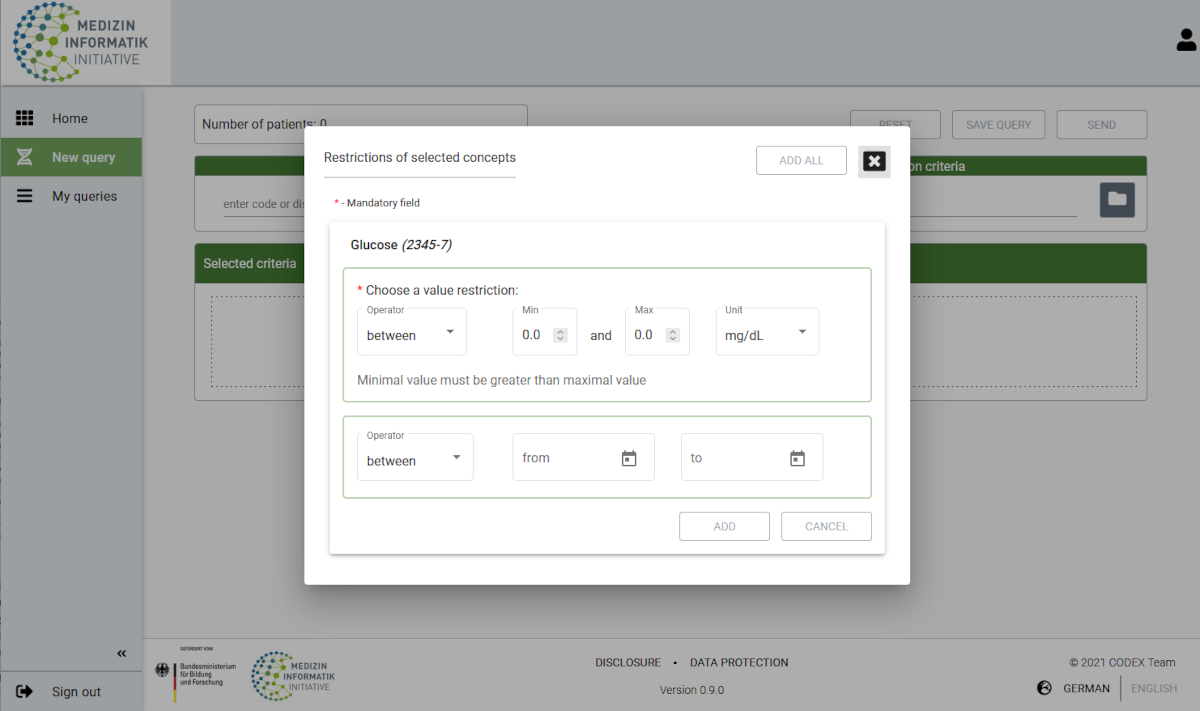
Pop-up window with the possibility to specify selected criteria.

After the initial selection of the criterion, a pop-up window opens offering the possibility to add further restrictions (Figure 3).

**Figure 4:**
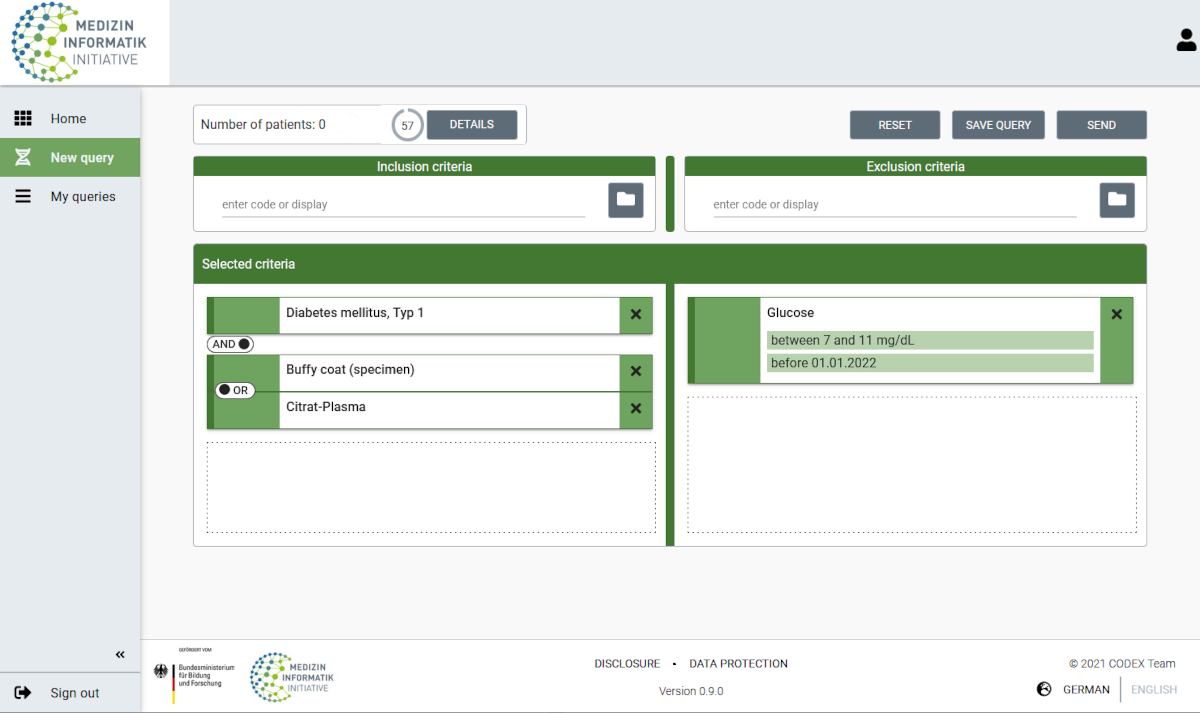
The query used to initiate the search process.

In addition to criterion-specific restrictions (eg, specification of a value range or the localization of the biospecimen), a temporal constraint is possible. The possibility to link the selected criteria using Boolean operators is offered as soon as the criteria have been finally added to the query. Once the desired query has been formulated, it can be executed (Figure 4).

As soon as the search query is processed, the result is displayed in the upper area under *Number of patients*. The *Details* option provides an overview of the breakdown of the cumulative result, although the data-providing hospitals remain anonymous.

### Study Flow

Interested participants were enrolled in the study after being recruited and provided with detailed information, including an informed consent form and a privacy statement. Upon receipt of the signed forms, an appointment was made to conduct the evaluation, which lasted approximately 60 minutes. After attending a brief welcome session and having been provided an overview of the study, the participants had to solve 3 tasks as part of an exploratory usability walk-through. The test leader protocolled the testing and the comments of the participants in a structured form. After the participants had completed the test tasks, we collected information regarding usability, demographic aspects, expertise, and so on, using the web-based survey tool SoSci Survey [12]. Subsequently, participants were able to provide their input on the various additional functions presented using mock-ups. Feedback on the acceptance and added value of these possible implementations was collected using a structured interview.

For backup reasons, the entire session was captured on Zoom (Zoom Video Communications, Inc) [13] using the videoconferencing platform’s recording function and stored in a password-protected cloud folder.

### Instruments

#### Tasks

The evaluation team had compiled 2 test tasks themselves to be able to cover the entire range of functions of the feasibility tool v2 as far as possible. Care was taken to ensure that these tasks reflected realistic requests and varied in their degree of complexity. Moreover, a third task was formulated based on a real-world request submitted during an MII workshop. While carrying out the tasks, the test participants were encouraged to express their thoughts aloud according to the thinking aloud method [14,15]. The aim of this method was to gather immediate feedback on the strengths and weaknesses of the tool. In addition, suggestions for improvement, if any, were noted. The correctness of the tasks was evaluated by checking whether all criteria were correctly selected and linked and led to the required query. A scoring system was used to determine the number of points for each task performed. The test tasks can be found in Multimedia Appendix 1.

#### Questionnaires

After completing the tasks, the test participants were asked to describe their immediate impression of the feasibility tool v2 and, in particular, to list positive and negative design aspects as well as make suggestions for improvement. Subsequently, they were asked to assess the usability of the query tool using the System Usability Scale (SUS). According to Brooke [16], the SUS is a standardized and validated questionnaire that allows a quantitative assessment of the usability of the tested systems. In addition to the SUS questions on general usability, 4 more questions focused on the usability of the category search. Furthermore, the test participants were asked to answer questions regarding personal details, expertise, and experience. The questionnaires that were used in the evaluation can be found in Multimedia Appendix 2.

#### Interview and Mock-ups

**Figure 5:**
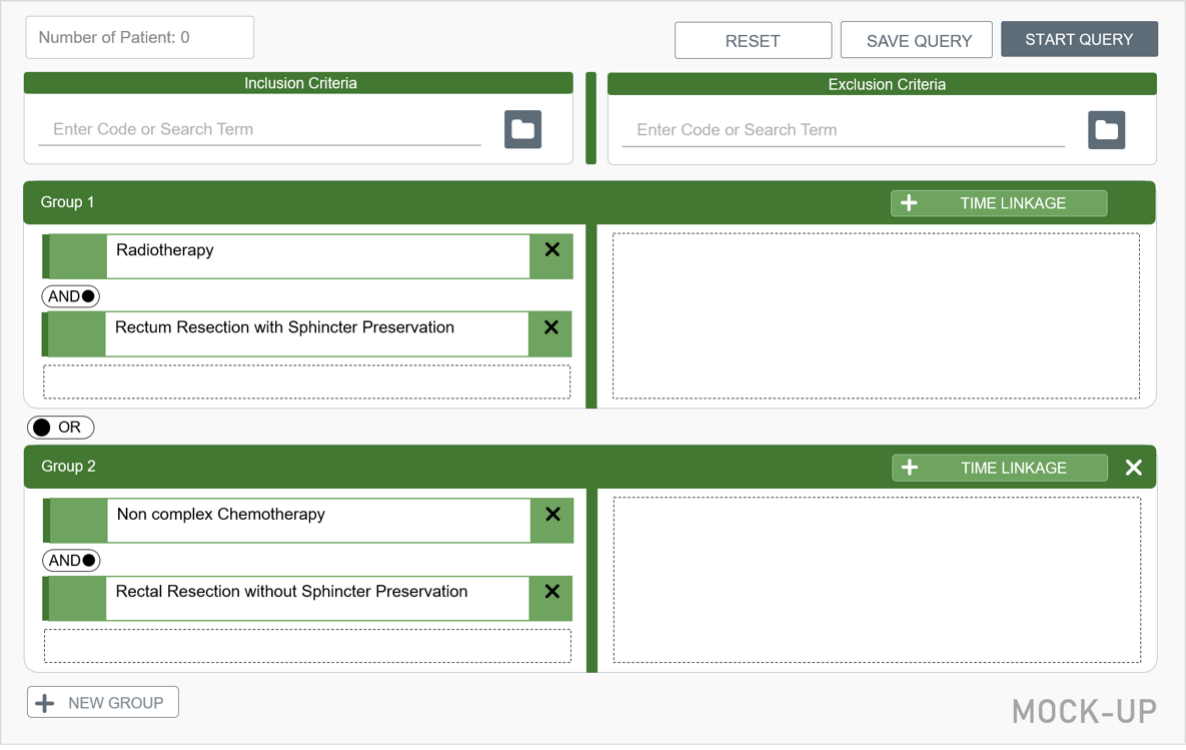
Mock-up for the additional feature groups.

**Figure 6:**
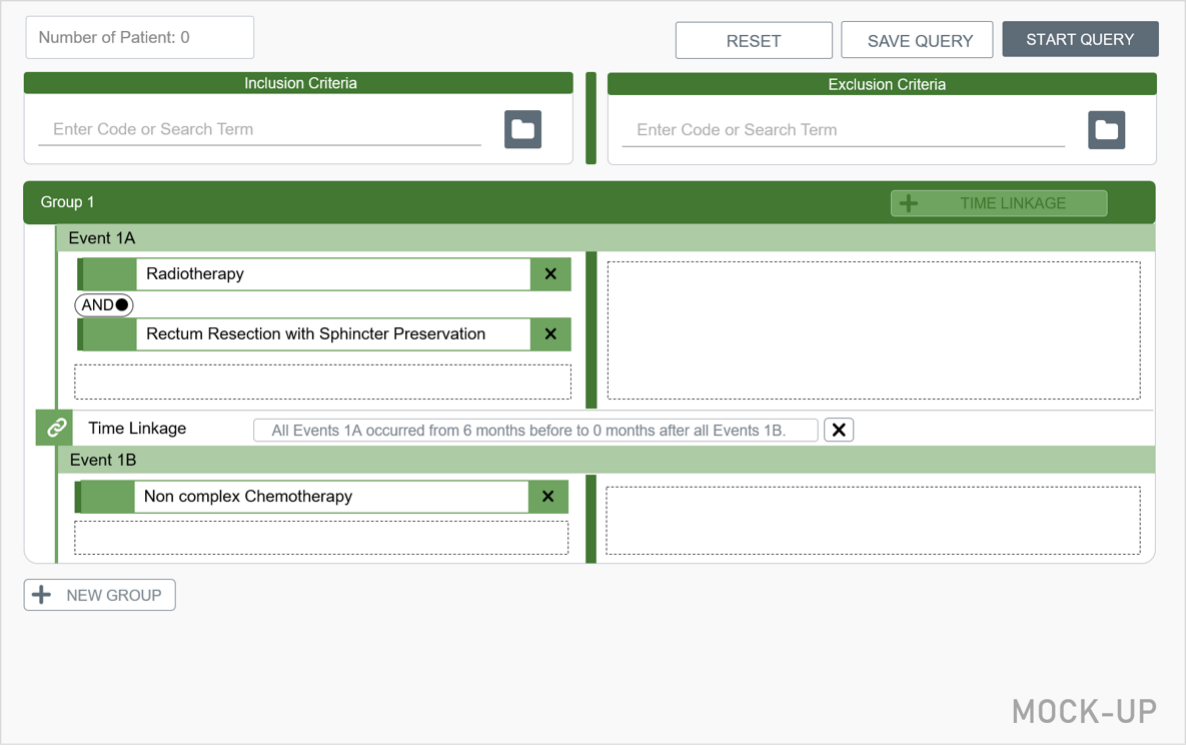
Mock-up for the additional feature Time Linkage.

A final interview block [17] served to determine user preferences regarding the implementation of new functions and whether this was congruent with the intended implementation. Mock-ups were created for the additional functions *groups* and *temporal dependencies* based on exemplary queries (Figures 5 and 6).

The corresponding interaction path was demonstrated to the test participants by the test leader for illustration purposes. On the basis of these mock-ups, the participants were asked to assess whether they perceived the approach as intuitive and, if not, what navigation path they would have expected. For the representation of *temporal dependencies* among the criteria or criteria groups, in the sense that, for example, conventional therapy was provided before an interventional procedure, the participants were asked whether they would see added value in this and how functional such a representation would have to be (in terms of the number of criteria that would have to be linked). Finally, the necessity to represent nested criteria—in terms of linking a criterion with another criterion, such as the International Classification of Diseases, Tenth Revision (ICD-10), diagnosis D43 (neoplasms of uncertain or unknown behavior of the brain and central nervous system) combined with the International Classification of Diseases for Oncology, version 3 (ICD-O-3), morphology 9383/1 (subependymoma)—was discussed with the participants. To find out the preferences of users, the test participants received an exemplary query to illustrate the problem. Although in its current state of development the tool did not offer the possibility to formulate this query in a single query, the test participants were motivated to express which approach they would have intuitively chosen or which functionality they would have expected to be able to formulate the query correctly.

### Data Analysis

#### Analysis of the Thinking Aloud Protocols

After the test sessions, the task processing protocols were checked for completeness, supplemented if necessary, and electronically documented. All positive and negative aspects of the tool were extracted from the protocols. Three usability and ontology experts categorized the problems separately as usability related or ontology related. The consensus decision was documented in a list. In cases where a sharp distinction between usability-related problem and ontology-related problem was not possible, these were grouped in a separate cluster. The negative aspects were additionally rated by 2 experts using the severity scale developed by Nielsen et al [18].

#### Task Success

The correctness of task processing was both evaluated globally and differentiated for the respective task steps using a self-developed scoring system (Multimedia Appendix 3). We analyzed the mean score achieved across all participants, the SD, and the accuracy rate in percentages.

#### Analysis of the Web-Based Questionnaire

Regarding the SUS, we applied a quantitative evaluation using the scoring method formulated by Brooke [16]. The responses provided to the additional questions related to the criteria search were summed up per item. For questions regarding the participant, a descriptive evaluation (frequencies, mean values, and SDs) was performed.

#### Analysis of the Interview on Additional Features

Analogous to the thinking aloud protocols, the feedback obtained during the interviews regarding the additional functionalities was recorded and documented electronically. The statements were subjected to a descriptive qualitative content analysis.

## Results

### Sample Description

The study cohort consisted of 22 test participants from 14 ABIDE partners. This corresponds to 92% (22/24) of the potential participants approached and thus comfortably exceeds the planned sample size of 14 test participants. The majority of the study cohort was composed of the younger age groups *25 to 34 years* (8/22, 36%) and *35 to 44 years* (11/22, 50%). Of the 22 participants, 9 (41%) were male, and 13 (59%) were female; in terms of profession, 7 (32%) were researchers, 4 (18%) had a biobanking background, 8 (36%) were IT professionals with a research background, and 3 (14%) were from other groups or did not specify. Work experience averaged 4.65 (SD 5.34) years. Participants declared no (10/22, 45%) or only some (12/22, 55%) prior experience with feasibility queries; prior experience with similar systems was reported by only 9 (41%) of the 22 participants. Whereas the test participants rated their IT knowledge as at least medium (medium: 9/22, 41%, and high: 15/22, 68%), the ratings on medical knowledge ranged from *very low* (2/22, 9%) to *rather low* (5/22, 23%) and *medium* (8/22, 36%) to *rather high* (7/22, 32%). The detailed characteristics of the study cohort are shown in Table 1.

**Table 1:** Detailed characteristics of the study cohort.

Variables		Values
**Age group (years), n (%)**		
	25 to 34	8 (36)
	35 to 44	11 (50)
	45 to 54	2 (9)
	55 to 64	1 (5)
**Sex** **(observed, not asked),** **n (%)**		
	Male	9 (41)
	Female	13 (59)
**Profession, n (%)**		
	Researcher	7 (32)
	Professional with biobanking background	4 (18)
	IT professional with research background	8 (36)
	Other	2 (9)
	Not specified	1 (5)
Work experience (years), mean (SD)		4.65 (5.34)
**Prior experience with feasibility queries, n (%)**		
	None	10 (45)
	Some	12 (55)
**Prior experience with similar systems, n (%)**		
	No	13 (59)
	Yes	9 (41)
**IT** **knowledge, n (%)**		
	Medium	9 (41)
	High	13 (59)
**Medical knowledge, n (%)**		
	Very low	2 (9)
	Rather low	5 (23)
	Medium	8 (36)
	Rather high	7 (32)

### Task Success

The effectiveness analysis (completeness and accuracy) showed that no participant managed to solve all the tasks correctly (in the sense of matching the model solution). Task 1a was successfully completed by half of the test participants (11/22, 50%). Task 1b displayed the best performance with a success rate of 100%. In task 2, of the 22 participants, 14 (64%) obtained the correct result. By contrast, only 1 (5%) of the 22 participants was able to solve task 3.

The accuracy analysis of the partial steps that had to be processed within the assignments based on the scoring system is presented in Table 2.

**Table 2:** Task success according to the scoring system.

Task	Maximum possible score	Mean score achieved (SD)	Accuracy rate, %
Task 1a	8	7.23 (1.28)	90.37
Task 1b	1	1.00 (0.00)	100
Task 2	8	7.64 (0.48)	95.50
Task 3	5	3.32 (0.87)	66.40

Of the maximum possible 8 points in task 1a and task 2, participants obtained an average of 7.23 (SD 1.28) points and 7.64 (SD 0.48) points, respectively. This corresponds to a success rate of 90.37% and 95.50%, respectively. Task 3 could only be completed correctly with an accuracy of 66.40%. With a maximum of 5 possible points, this corresponds to an average of 3.32 (SD 0.87) points scored. In task 1a, the major source of error was the choice of diagnosis (8/22, 36%). Instead of choosing “Essential (primary) hypertension,” participants often selected another characteristic containing the term “hypertension” (eg, “Hypertension [hypertensive disease]”). The same potential for error was present in task 3 for both criteria (“Vancomycin” [selected by 18, 82% of the 22 participants] and “treated in intensive care” [selected by 7, 32% of the 22 participants]) being searched. Less frequently, errors occurred because of an incorrect *AND* or *OR* used to link the criteria (5/22, 23%) or when entering time constraints (5/22, 23%).

### Global Assessment of Usability (SUS Score)

Textbox 1 shows the respective mean scores of the SUS items. The SUS score of the feasibility tool v2 calculated across all participants was 81.25 (SD 13.42) on a scale of 0 to 100.

**Summary of the System Usability Scale–item results based on a scale ranging from 1 (strongly disagree) to 5 (strongly agree).**

System Usability Scale item and mean (SD) values

- I think that I would like to use this query tool frequently: 4.6 (0.5)
- I found the query tool unnecessarily complex: 1.6 (1.0)
- I thought that the query tool was easy to use: 4.3 (0.8)
- I think that I would need the support of a technical person to be able to use this query tool: 1.9 (1.0)
- I found the various functions in this query tool were well integrated: 4.1 (0.7)
- I thought there was too much inconsistency in this query tool: 1.8 (0.9)
- I would imagine that most people would learn to use this query tool very quickly: 4.3 (0.8)
- I found the query tool very cumbersome to use: 1.5 (1.0)
- I felt very confident using the query tool: 3.8 (0.9)
- I needed to learn a lot of things before I could get going with this query tool: 1.8 (0.7)

The mean SUS score of test participants classified as *IT professionals with research background* was 88.75 (SD 8.00), which, in comparison with the mean SUS scores of the primary user groups *researchers* (mean 78.13, SD 9.00) and *professionals with biobanking* *background* (mean 70.00, SD 13.46), was slightly higher.

**Figure 7:**
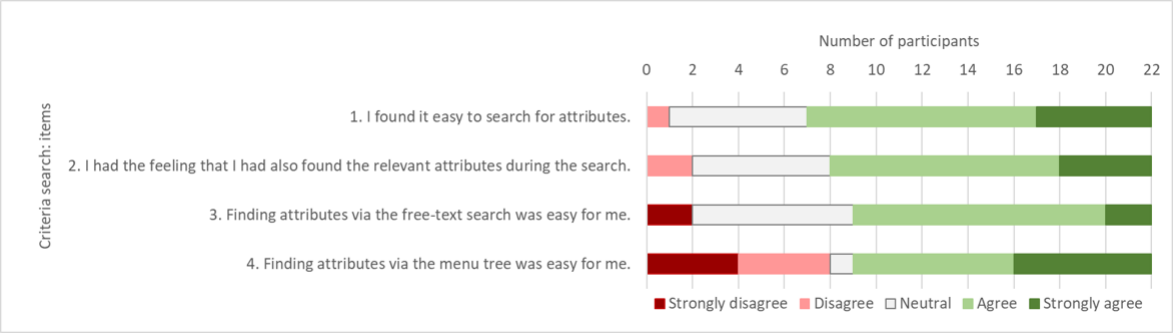
Rating of the additional items regarding the findability of criteria.

The evaluation of the findability of criteria—based on the questions formulated in addition to the SUS scores—by the study participants indicates that the search for criteria was perceived as easy. Participants found that searching via the category tree tended to be more difficult than via the free-text search. More than half of the participants (14/22, 64%) had the impression that they could easily find the relevant criteria to solve the test tasks. Figure 7 shows the rating of the 4 additional items.

### Usability Aspects Identified

#### General Aspects

The analysis of the thinking aloud protocol revealed that the majority of the participants (13/22, 59%) assessed the user interface of the feasibility tool v2 as simple to use and intuitive. Searching for criteria using the free-text search was frequently emphasized as a helpful feature. Moreover, the clarity of the user interface and visual separation of the inclusion and exclusion criteria were highlighted as particularly positive. The switch button that makes it easy to change *AND* to *OR* was considered a well-integrated solution.

In addition to the positive aspects, 39 usability problems were identified and classified using the severity scale developed by Nielsen et al [18] as follows: 5 (13%) were classified as *usability catastrophes*, 8 (21%) as *major usability problem*s, 12 (31%) as *minor usability problem*s, and 14 (36%) as *cosmetic problems*.

Among the 5 usability catastrophes was that the free-text search bar was not easily located since the free-text input fields are grayed out indicating inactivity. In addition, the identification of relevant criteria in the results list of the free-text search was partly perceived as difficult, first because of the missing labeling of the code type and second because of the absence of traceability of the criteria path. Furthermore, the restriction of the time period with the operator *between* led to critical usability situations because this operator does not implicitly process the time specification when only 1 date is entered for a *before* or *after* query. The missing display of the codes when the selected criteria appear in the search interface also resulted in ambiguity.

The usability catastrophes and major usability problems are visualized in Multimedia Appendix 4, and the associated optimizations are suggested.

#### Ontology-Specific Aspects

The study participants assessed the orientation at the upper level of the category tree as good. In addition, it was observed that the orientation at lower levels was perceived as comprehensible by the test participants if they had background knowledge about the criteria. Overall, most of the participants (14/22, 64%) found it quite easy to identify relevant criteria as shown in Figure 7 (item 2). However, it was often observed that the display of identical or similar criteria in the free-text results list led to uncertainty in identifying relevant criteria. This was partly because of the lack of a path display, as described in the previous subsection, and partly because of the complexity and ambiguity of the ontology (eg, criteria such as *glucose*, *glucose/BK*, and *glucose/blood* have identical paths).

The mixed use of German and English terms—predetermined by the MII core data set—was perceived as cumbersome by some test participants and led to comprehension problems. The sorting of the criteria in the category tree was criticized at several points, and preferred alternatives were suggested; for example, some of the participants (4/22, 18%) wanted the criteria to be ordered alphabetically, whereas others (2/22, 9%) preferred sorting by relevance. Furthermore, criteria with the designation *Other (...)* were expected to be placed at the end of the list. When searching for *female patients*, it was not clearly apparent that sex had to be selected to add the characteristic *female*. Test participants expected the characteristic *female* to be selected directly in the category tree. Furthermore, some of the test participants (3/22, 14%) found the category tree to be textually overloaded.

### Feedback on Additional Features

With regard to the additional features presented in the supplementary mock-ups, the interview analysis revealed that the implementation of the group function was considered successful and intuitive by almost all of the test participants (21/22, 95%). However, it was also pointed out that the *NEW GROUP* button should be made clearer and more obvious and that the assignment of characteristics to the respective desired groups should be made as simple as possible, requiring only a few clicks.

The option to link subgroups within a group in terms of temporal dependencies was perceived as rather complex. In principle, the function is considered useful because questions with temporal dependencies occur frequently, especially in the oncology field. However, the presented implementation of the function was still perceived as not very intuitive. Possible improvements could involve providing (1) a stronger emphasis of the button *TIME LINKAGE*, (2) context-specific information via mouse-over text, (3) a link to a brief *How to* section, and (4) a tutorial explaining this feature.

The discussion on the depth of criteria nesting provided a heterogeneous picture. Regarding the intuitive approach, the recorded solutions varied from the entry of individual criteria and the formation of groups to the desired possibility of assigning criteria directly to other related criteria (eg, assigning the criterion *subcutaneous* to the criterion *insulin*). The majority of the participants (14/22, 64%) would have solved the example task via groups, but this can only serve as a rough orientation because the task in the form set could only be solved theoretically and could not be worked out using the tool.

## Discussion

### Overview

The rationale for this work was to simultaneously develop and assess the feasibility tool v2 regarding usability and to evaluate the comprehensibility of the underlying ontology with regard to the findability of criteria.

### Discussion of Methods

Thinking aloud tests are an established method for formative evaluations to identify usability problems and their causes early in the development process and have been applied several times in the clinical field for usability evaluation of query builders [3,8,19]. However, because of their qualitative nature, thinking aloud tests do not allow a quantitative evaluation of usability. This methodological disadvantage was compensated for by using the SUS to obtain an overall statement about how well the design of the feasibility tool v2 has succeeded. The SUS is a standardized instrument that can be used for any type of system, and it can provide valid insights into whether and to what extent usability problems exist [20]. The SUS has also been used in clinical settings for query builders [19,21].

In addition, we conducted user interviews, which are fundamentally well suited to elicit user desires and insights and have been applied several times for usability evaluations [22,23]. As our goal was not to perform statistical analyses but to collect preferences and suggestions for improvement, this method was an adequate choice.

Usability tests were conducted with a sample of 22 participants. This number is sufficient from the point of view of conducting (1) the thinking aloud test, which requires a minimum of 3 to 5 test participants [24]; (2) the SUS, which requires approximately 12 persons to reach an apparent asymptote [25]; and (3) user interviews, which require approximately 12 persons for researchers to obtain sufficient information about user problems [26].

Overall, the combination of methods allowed us to obtain a very diverse picture of user views and identify important usability issues that would need to be addressed in the next iteration. Furthermore, this combination of methods was easy to apply without the need for any special application knowledge and could be performed within a reasonable amount of time to obtain ideas for further developments very promptly.

### Discussion of Results

The evaluation of the usability of the feasibility tool v2 indicated a good degree of user-friendliness. The quantitative evaluation of the SUS questionnaire also confirmed the impression gathered through user feedback. In comparison with the previous version of the prototype, it can be stated that the critical usability problems identified in the evaluation by Sedlmayr et al [8], such as the difficulty in distinguishing between inclusion and exclusion criteria or the unclear linkage using the Boolean operators, could be successfully solved and occur only in negligible numbers. There were 5 usability catastrophes in the feasibility tool v2 and 8 major problems; in comparison, there were 8 usability catastrophes and 4 major problems in the previous iteration. In this respect, there were individual improvements in usability; however, overall, there is still a need for adjustments. No comparisons can be made regarding the SUS score because the previous version was evaluated using a different set of methods (user interviews instead of web-based questionnaires), which is not unusual in iterative user-centered development [27].

Comparing the feasibility tool v2 with similar tools, it can be stated that it performs relatively well. With a SUS score of 81.15, the feasibility tool v2 performed better than the query tools Informatics for Integrating Biology and the Bedside (i2b2) [28] (SUS score=59.83); ATLAS, developed by Observational Health Data Sciences and Informatics (OHDSI) [29] (SUS score=27.81); and the GBA Sample Locator [30] (SUS score=77.03), whose user-friendliness was examined in a study published in 2021 [3]. In addition to these positive results, it must be mentioned that there is still potential for improvement. Along with making further adjustments in the area of design and refinements in navigation, the focus now is on the new features and underlying ontology.

Although the group function is technical and graphical rather easy to implement, the temporal link is more complex. Methods for technical as well as graphical implementation can already be found in the literature [31]; for example, search tools such as the aforementioned i2b2 and ATLAS take a text-based approach to display, and there are also graphical solutions such as QueryMarvel [32]. Challenges in this regard arise primarily in the technical implementation as well as in a matching intuitive presentation that should enable error-free use. The aforementioned approaches, in conjunction with the feedback from the evaluation study, will play a vital role in the deliberations that will be conducted for the next iteration process.

We also discovered that the underlying ontology has a crucial impact on the usability and acceptance of a feasibility tool. This was particularly evident in the direct comparison between the extended version with the comprehensive MII core data set [10] and the previous version with the rather lean GECCO [9]. Although there were hardly any difficulties in selecting the criteria searched for in the feasibility tool v1 [8], it was observed in the feasibility tool v2 that the search required extra time because of the more extensive ontology. It should be noted that navigation through the category tree as well as via the free-text search depends on the existing background expertise of the user. Participants with knowledge of medical terminology found it easier to navigate the category tree, whereas participants who were not familiar with relevant classifications, such as ICD-10 codes or Logical Observation Identifiers Names and Codes (LOINC) codes, had to resort to the trial-and-error method at times. This observation is also reflected in the results of the SUS score evaluation by the professionals; for example, study participants who had a medical background rated the tool better (SUS score=78.13) than those who had a biobanking background (SUS score=70.00) and tend to come from a natural science background and are unfamiliar with diagnostic and laboratory codes. This is also in line with the findings from the study comparing the 3 feasibility platforms [3], which strongly suggest that tools with more functionalities and a more extensive ontology have a harder time providing an intuitive interface. This confirms the appropriateness of our approach, which involves conducting regular evaluations based on the user-centered design process [33], thus enabling us to directly incorporate user feedback into further iterations.

### Limitations

Despite the efforts we made to apply a real-world approach to the study design to obtain meaningful results for the subsequent development steps, our work includes some limitations. First, it should be mentioned that test participants were recruited for the study at sites that were ABIDE project partners. Nevertheless, care was taken to ensure that the participants were not directly involved in the project work so that they could provide an unbiased evaluation. Another aspect that could have contributed to selection bias is the fact that the study was conducted via Zoom. This method saves time and resources, but it lacks the advantages offered by a standardized test environment, although the literature shows that remote testing can be expected to produce results similar to those of laboratory testing and is an equally good method for usability testing [34,35]. As our study was conducted remotely, it is possible that mainly people with basic IT skills signed up to participate. In fact, all participants indicated that they had at least an intermediate understanding of IT. Thus, we cannot eliminate the possibility that we lack input from people who have no or little general IT expertise. Nevertheless, it can be assumed that this group of people will not be among the main users of the ABIDE feasibility tool.

Another limitation is that a prototype was evaluated. On the one hand, this had the consequence that neither test data nor real data were connected; thus, no realistic results could be provided after the query was sent. As this is only a small aspect, and the focus was on the general usability of the tool, it can be assumed that this factor is negligible. On the other hand, because the prototype did not contain all functionalities, the envisaged additional functions could only be presented in the form of mock-ups. In this way, the analysis of the navigation path and usability was limited. However, because the evaluation took place during development, we see it as an advantage that the planned implementation could first be tested using the mock-ups before any programming work was done. According to the feedback, a revised version of the mock-ups can now be created, which can then serve as a reference for the development team.

We would like to point out that, under certain circumstances, the different ways of presenting scenarios (2 tasks in tabular format and 1 in free-text format), test execution time, and the current fatigue state of the participants could have had a possible influence on the results. However, we conducted an exploratory study with a focus on collecting suggestions for improvement for the next iteration and not a classical experiment where it is common to perform a confounder analysis.

The exclusive use of the SUS as a standardized questionnaire could be perceived as an additional limitation. Although the SUS has been used previously to evaluate ontologies, it had to be adapted for this purpose [36]. Consequently, a scale for assessing the usability of ontologies— the Ontology Usability Scale [37]—was developed, which adapts the SUS items and tailors them to ontologies. We have refrained from such a detailed evaluation of the ontology and limited ourselves to 4 items. We specifically focused on usability in the sense of ease of use, meaning that an extended consideration of the ontology would have exceeded the time frame of our study. Moreover, and this is probably the more essential point, we have no immediate influence on the ontology because it is a direct representation of the terminology used in the MII core data set, and because this is the responsibility of other working groups outside the ABIDE project, we cannot optimize it independently based on the results. Nevertheless, it was our intention not to completely disregard the ontology to identify usability problems that occur because of the underlying ontology. If these cannot be compensated by changes in the graphical user interface, we will forward the documented problem areas as the basis for discussion to the responsible persons.

### Conclusions

The findings from the evaluation indicate that the investigated prototype of the feasibility tool v2 has good usability. The global SUS score of 81.25 can be rated as *good*. The collected feedback supports this result because the tool was frequently described as intuitive and user friendly. However, the analysis of user feedback also revealed areas that need revision. For our next development iteration, for example, we see potential for optimization above all in the display of the search functions, the unambiguous distinguishability of criteria and visibility of their associated classification system, and the implementation of the temporal linking of criteria for which recommendations for improvement will be developed. Furthermore, the findings on the comprehensibility of the ontology will be fed back to the responsible departments so that corrections can be made here as well. Overall, it can be stated that the combination of different tools used to evaluate the feasibility tool v2 provided a comprehensive view of its usability. As a development-accompanying method, we can recommend this in the planning and implementation of similar projects to be able to closely control the course of development and correct it if necessary.

## Appendix

Multimedia Appendix 1 Test tasks of the usability evaluation of the Aligning Biobanking and Data Integration Centers Efficiently feasibility tool v2.

Multimedia Appendix 2 Questionnaires of the usability evaluation of the Aligning Biobanking and Data Integration Centers Efficiently feasibility tool v2.

Multimedia Appendix 3 Scoring system for the assessment of the correctness of task processing.

Multimedia Appendix 4 Usability catastrophes and major usability problems (visualization and optimization).

## Abbreviations

ABIDE: Aligning Biobanking and Data Integration Centers Efficiently
CODEX: COVID-19 Data Exchange Platform
DIC: data integration center
FDPG: Research Data Portal for Health (Forschungsdatenplattform für Gesundheit)
FHIR: Fast Healthcare Interoperability Resources
GBA: German Biobank Alliance
GBN: German Biobank Node
GECCO: German Corona Consensus Data Set
i2b2: Informatics for Integrating Biology and the Bedside
ICD-10: International Classification of Diseases, Tenth Revision
ICD-O-3: International Classification of Diseases for Oncology, version 3
LOINC: Logical Observation Identifiers Names and Codes
MII: Medical Informatics Initiative (Medizininformatik-Initiative)
OHDSI: Observational Health Data Sciences and Informatics
SUS: System Usability Scale
