# Supplementary material for: Empowering Researchers to Query Medical Data and Biospecimens by Ensuring Appropriate Usability of a Feasibility Tool: Evaluation Study
Source: JMIR Hum Factors. 2023 Apr 19;10:e43782. doi: 10.2196/43782 (PMC10157450; doi:10.2196/43782)
Supplement: Multimedia Appendix 1 [file humanfactors_v10i1e43782_app1.pdf]

Evaluation of the ABIDE feasibility tool – Test tasks

While working on the test task, please think aloud and say at any time**:**

- What would you do now?
- How would you proceed with this?
- What works well in the process /What do you like about the ABIDE Feasibility Tool?
- What is problematic for you in this process and why?

Test task 1

Task 1a) Please determine the **number of those patients** who have the following characteristics:

Inclusion criteria:

- Female patients
- All available consents for
  - the use and collection of clinical data
  - the use of biosamples
  - Re-contacting posibilities
- Essential (primary) hypertension

Exclusion criteria:

- Administration of diuretics

Task 1b) Now save your query.

Test task 2

Task 2)

Please determine the **number of those patients** who have the following characteristics:

Inclusion criteria:

- Patients with diabetes mellitus, type 1
- Biosamples: serum **or** citrated plasma

Exclusion criteria:

- Glucose value in blood lower than 160mg/dL in the period 01.01.2021 - 30.04.2021

Test task 3

Task 3)

Please determine the **number of patients** for whom there is at least one documented administration of the antibiotic vancomycin and who received intensive care treatment at the site prior to 01.01.2021.
