# Supplementary material for: Empowering Researchers to Query Medical Data and Biospecimens by Ensuring Appropriate Usability of a Feasibility Tool: Evaluation Study
Source: JMIR Hum Factors. 2023 Apr 19;10:e43782. doi: 10.2196/43782 (PMC10157450; doi:10.2196/43782)
Supplement: Multimedia Appendix 2 [file humanfactors_v10i1e43782_app2.pdf]

Evaluation of the ABIDE feasibility tool – Questionnaires

System Usability Scale

Statement	Strongly disagree	Disagree	Neutral	Agree	Strongly agree
I think that I would like to use this tool frequently.					
I found the tool unnecessarily complex.					
I thought the tool was easy to use.					
I think that I would need the support of a technical person to be able to use this tool					
I found the various functions in this tool were well integrated.					
I thought there was too much inconsistency in this tool.					
I would imagine that most people would learn to use this tool very quickly.					
I found the tool very cumbersome to use.					
I felt very confident using the tool.					
I needed to learn a lot oft hings before I could get going with this tool.					

Additional questions related to the criteria search

Statement	Strongly disagree	Disagree	Neutral	Agree	Strongly agree
I was able to find my way properly when searching for criteria.					
I had a hard time finding criteria using the free text search.					
I had the feeling that I found the relevant criteria during the search.					
I perceived the finding of criteria via the category tree as difficult.					

Questions about characteristics of the study cohort

**Age group**	
What age group do you belong to?:<br>• Younger than 25 years<br>• 25 to 34 years<br>• 35 to 44 years<br>• 45 to 50 years<br>• 55 to 64 years or<br>• 65 years and older	☐ Younger than 25 years<br>☐ 25 to 34 years<br>☐ 35 to 44 years<br>☐ 45 to 54 years<br>☐ 55 to 64 years<br>☐ 65 years and older<br>☐ Not specified
**Professional Groups**	
To which professional group do you belong:<br>• Researchers<br>• Personnel with biobanking background<br>• IT specialist in research environment<br>• Others	☐ Researchers<br>☐ Biobanker/in Personnel with biobanking background<br>☐ IT specialist in research environment<br>☐ Others (please indicate):<br>____<br>☐ Not specified
**Work experience**	
How long have you worked in your current position?	☐ In years: __________<br>☐ Not specified
**Prior experience with feasibility queries**	
How would you rate your experience with requesting case numbers for clinical trials?	☐ No experience<br>☐ Some experience<br>☐ Lot of experience<br>☐ Not specified
**Prior experience with similar systems**	
Have you used other similar systems in the past?	☐ No<br>☐ Yes/ which? (please indicate):<br>____<br>☐ Not specified
**IT knowledge**	
How would you rate your computer skills/knowledge?	☐ Low: I find many systems difficult to use.<br>☐ Medium: I get along well with most systems.<br>☐ High: I am very experienced and technically skilled.<br>☐ Not specified
**Medical knowledge**	
How would you rate your medical knowledge?	☐ Very low<br>☐ Rather low<br>☐ Medium<br>☐ Rather high<br>☐ Very high<br>☐ Not specified
