# Supplementary material for: Empowering Researchers to Query Medical Data and Biospecimens by Ensuring Appropriate Usability of a Feasibility Tool: Evaluation Study
Source: JMIR Hum Factors. 2023 Apr 19;10:e43782. doi: 10.2196/43782 (PMC10157450; doi:10.2196/43782)
Supplement: Multimedia Appendix 3 [file humanfactors_v10i1e43782_app3.pdf]

Evaluation of the ABIDE feasibility tool – Scoring system for the assessment of the correctness of task processing

General notes

- If it is obvious that the user forgot to start the query, then the point is still given, or, if a query was started at least once, then this is considered as correctly executed for all tasks.
- If a substep was solved in a different way e.g. via exclusion, but is correct in content, then the point is still given.

Task 1a

Points	Substep	Special cases
1	Female patients	
1	Use and collection of clinical data<br>"Collection, processing and scientific use (items 1.1 - 1.3)"	
1	Use of biosamples<br><u>REQUIRED:</u> "Biosamples: consent for collection, storage, and scientific use (items 3.1-3.3)“<br><u>Optional:</u> "Biosamples: extraction of small additional quantities (item 3.2)“	
1	Re-contacting posibilities: At least one of the following two criteria:<br>"Re-contact; additional scientific questions,… (item 4.1)“<br>"Re-contact: information about additional medical findings (item 4.2)“	
1	Essential (primary) hypertension	„Essential hypertension, unspecified“ does not give a point
1	Diuretics	
1	Correct linking of the criteria	
1	Running the query	
Total: 8 points		

Task 1b

Points	Substep	Special cases
1	Saving the query	
Total: 1 point		

Task 2

Points	Substep	Special cases
1	Diabetes mellitus, type 1	
1	Serum<br>„Serum specimen (specimen)“	„available“ can - but does not have to be ticked
1	Citrated plasma<br>„Plasama specimen with citrate (specimen)”	„available“ but does not have to be ticked
1	Glucose/blood	"Glucose (2345-7)" and "Glucose venous (41652-9)" also gives a point
1	< 160 mg/dL	Input with operator "between" instead of with "less" also gives a point
1	Between 01.01.2021 to 30.04.2021	
1	Correct linking of the criteria	
1	Running the query	
Total: 8 points		

**Task 3**		
Points	Substep	Special cases
1	All three vancomycin listed under Medication	• If the vancomycin laboratory finding is added as a fourth characteristic (in addition to the other three, then there is no point given<br>• If an additional time restriction was made with vancomycin, then there is no point given
1	Intensive medical treatment<br>REQUIRED: "Intensive care treatment“<br>Optional: "Intensive medical complex treatment", ... or similar.	
1	Prior to 01.01.2021	If operator was not changed and e.g. the input was made via "between", then there is no point given
1	Correct linking of the criteria	If multiple vancomycins have been selected, they must be linked with OR
1	Running the query	
Total: 5 points		
