# Supplementary material for: Empowering Researchers to Query Medical Data and Biospecimens by Ensuring Appropriate Usability of a Feasibility Tool: Evaluation Study
Source: JMIR Hum Factors. 2023 Apr 19;10:e43782. doi: 10.2196/43782 (PMC10157450; doi:10.2196/43782)
Supplement: Multimedia Appendix 4 [file humanfactors_v10i1e43782_app4.pdf]

Identified *usability catastrophes* and *major usability problems*

F = Frequency ; Total number of subjects: 22

<table>
<tr><th>Identified usability problem</th><th>Optimization recommendation</th><th>F</th></tr>
<tr><td colspan="3">USABILITY CATASTROPHE</td></tr>
<tr><td>

1) Finding the free-text search

The free text search was not found directly (either only with a hint from the test administrator or only very late). The dialog box that opens when clicking into the free text search was perceived as disturbing and confusing, because nothing has been entered yet and because of the graying out the free text field was perceived as inactive.

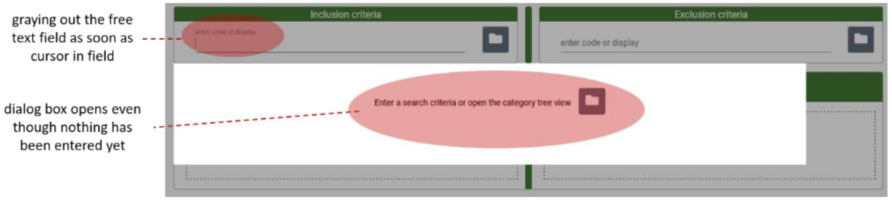

</td><td>

• Remove the dialog box
• Neither the free text field nor the rest of the screen should be grayed out.

</td><td>10</td></tr>
<tr><td>

2) Meaning of the codes unclear

The subjects often did not know the meaning of the codes. This led to uncertainty in the identification of relevant features.

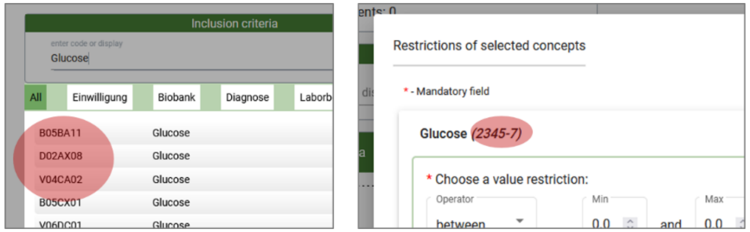

</td><td>

• Provide explanation different types of codes on help page
• Provide code with info text, which is explained in more detail on help page

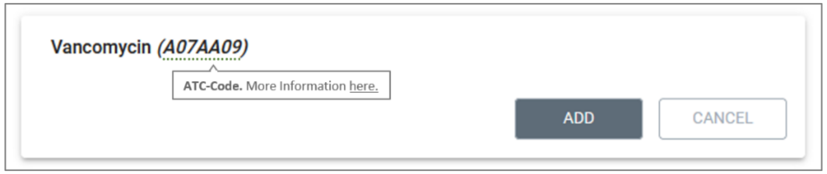

</td><td>11</td></tr>
<tr><td>

3) Missing display of the path of the criteria in the free-text search

For the subjects it was not obvious to which path the criteria in the result list of the free-text search belonged. This led to uncertainty in the identification of relevant features.

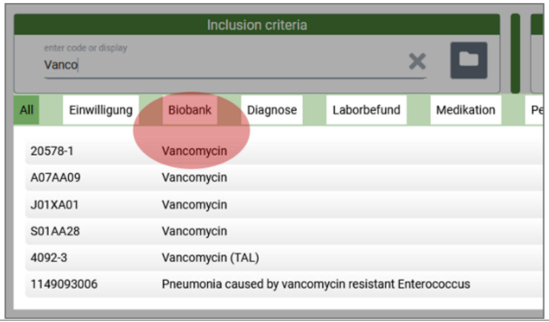

</td><td>

• Display the corresponding parent category and the last branch of the category tree in the results list of the free-text search

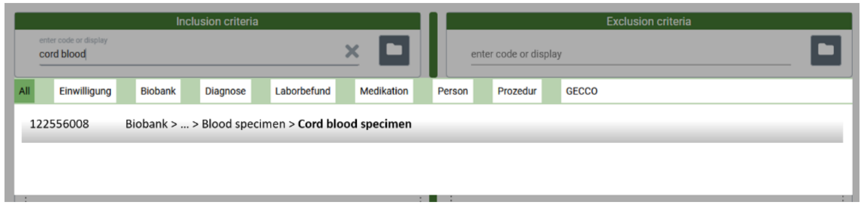

</td><td>10</td></tr>
</table>

4) Missing input option in the selection of the time period with the operator "between"<br>Subjects tried to enter the time restriction "before 01.01.2021" with the operator "between" instead of the operator "before" by entering only one date. However, the prototype executes a correct query until two dates are entered with the "between" operator.<br>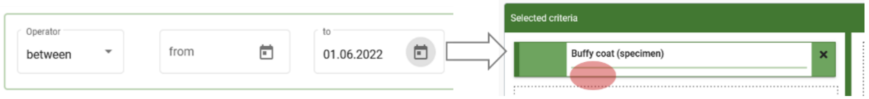	• Provide the input option: "one date input with the between operator"	5
5) Missing code display by the selected criteria display<br>The associated codes for the criteria are not displayed by the "Selected criteria display". If criteria with identical names are added, the absence of the codes means that no difference between the criteria is visible in the selected criteria display.<br>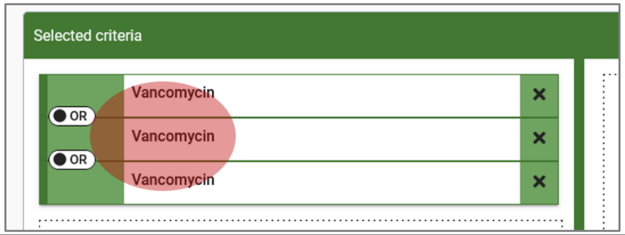	• Display the corresponding codes to the selected criteria in the selected criteria display<br>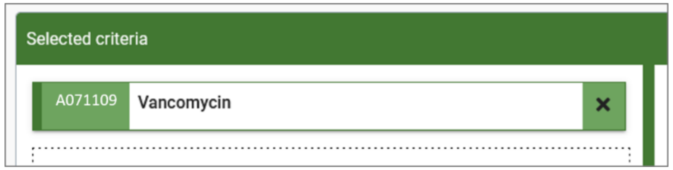	1
MAJOR USABILITY PROBLEMS		
1) Reset the query via the menu item "new query" is not possible<br>The functionality to reset the current query via the "New query" menu item in the left menu bar does not work.<br>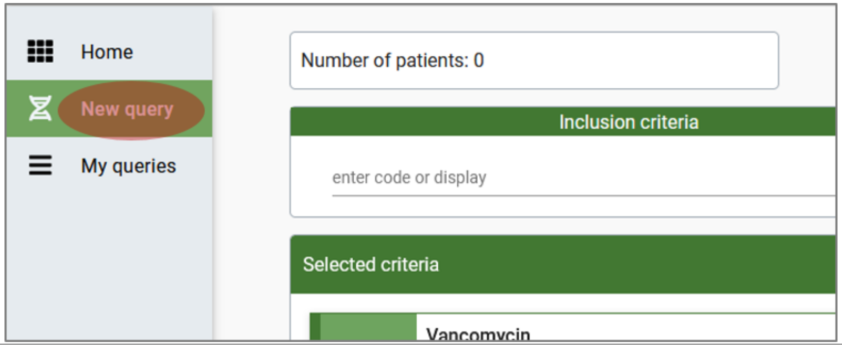	• Provide reseting the the query via the menu item "New query"	11
2) Display of limited number of hits in free-text search<br>Only the 20 most suitable hits are displayed in the free-text search. Subjects are irritated that they cannot find all criteria via the free-text search, which they have found via the category tree search.	• Offer the possibility to load additional results<br>• For example via button "more results" at the end of the result list	1
3) No display of related terms in free-text search	• Enable fuzzy search: Display similar hits	3

No hits are displayed for certain terms such as citrate plasma. No related terms are displayed.		
4) Free-text search field not recognizable as such<br>Sometimes subjects typed criteria into the input field and assumed it was thus added to the selected criteria. They did not realize that the field is a search field and not an input field.<br>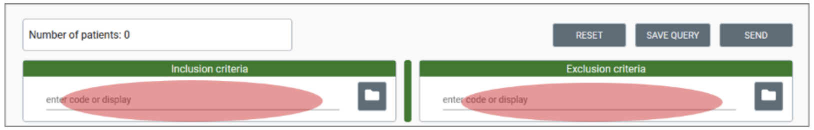	• Make free-text search field more recognizable as such for example with magnifying glass and frame around the search field<br>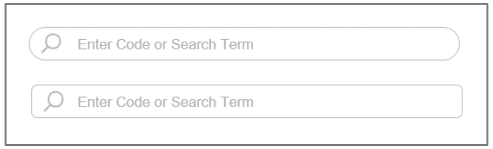	2
5) Process of adding and restricting criteria not clear<br>The subjects expected to add criteria by clicking the "Add" button, not being aware that they would have to add the criteria finally in the pop-up dialog (even though no restriction was to be made). They already expected adding at the first "Add" button.<br>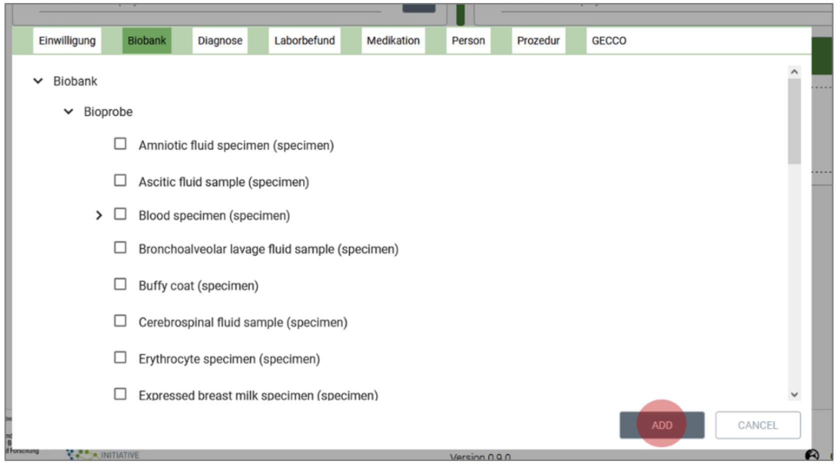	• Make process of adding and restricting criteria more clear<br>• For example: Change the button name: Rename the first "Add" button to "Next"<br>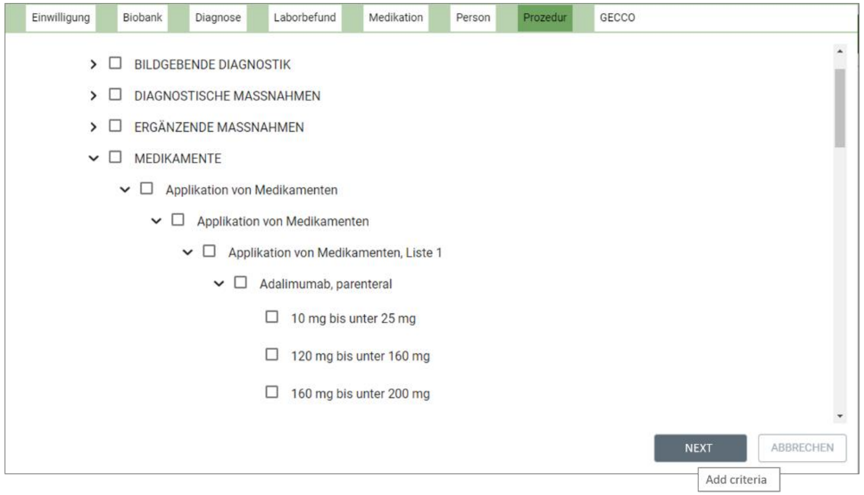	5
6) Finding the category tree search / No indication which search is active<br>The category tree search was partially not found. The folder button was overlooked because it is positioned in a less prominent place. Subjects could not clearly see whether the free-text search or the category tree search was currently active. (In addition, the graying out of the screen also plays a role here).	• Position folder button in front of free-text field<br>• Visual highlighting which search is currently active<br>Criteria tree search active:<br>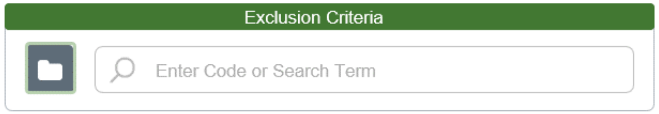<br>Free-text search active:<br>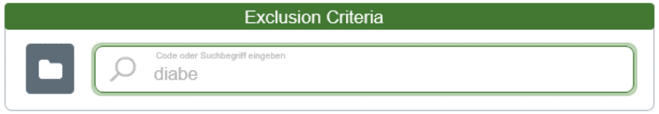	3

<table>
<tr>
<td>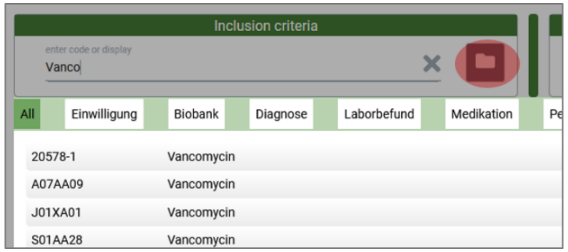
</td>
<td></td>
<td></td>
</tr>
<tr>
<td>7) Distinction between optional fields and mandatory fields not clear

Subjects were not always sure whether the input fields in the "Pop-up: Restriction of criteria" were mandatory or optional. The display of input fields suggests that the period is a mandatory field (see figure).
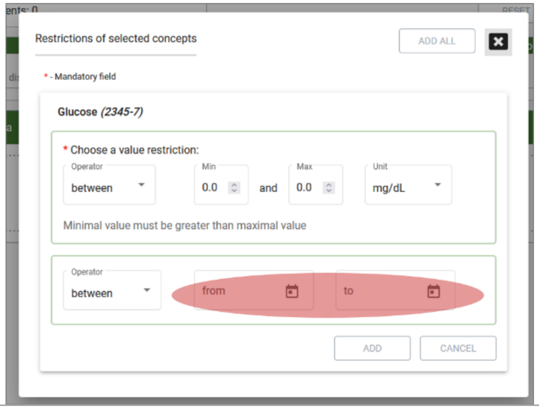
</td>
<td>• Optional fields should be initially collapsed by default so that the input fields are not visible
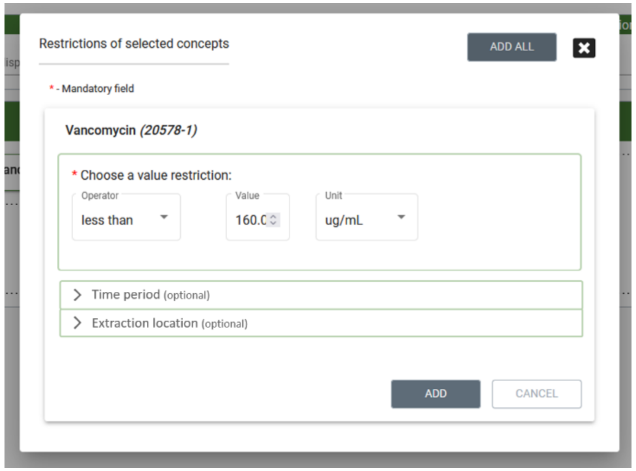
</td>
<td>9</td>
</tr>
<tr>
<td>8) No button for deleting criteria restriction (time restriction)

If subjects made an incorrect time constrain by selecting a criteria, they sometimes deleted the entire criteria from the criteria list because there is no explicit functionality to reset the constrain or deactivate it when it is not needed.
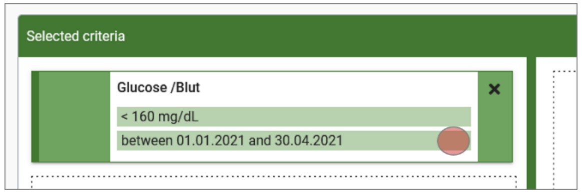
</td>
<td>• Allow "no specification" to be selected for the time restriction
• In addition, the general deactivation of constrains and restrictions should be implemented, for example with an "X"-button displayed next to the constrain
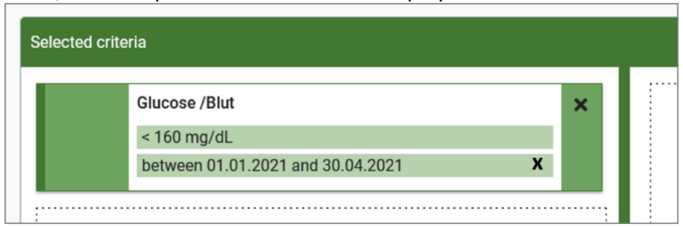
</td>
<td>3</td>
</tr>
</table>
